# Electronic Cigarettes and Oral Health

**DOI:** 10.1177/00220345211002116

**Published:** 2021-03-25

**Authors:** R. Holliday, B.W. Chaffee, N.S. Jakubovics, R. Kist, P.M. Preshaw

**Affiliations:** 1 School of Dental Sciences, Faculty of Medical Sciences, Newcastle University, Newcastle upon Tyne, UK; 2 School of Dentistry, University of California, San Francisco, CA, USA; 3 School of Dentistry, University of Dundee, Dundee, UK

**Keywords:** electronic nicotine delivery systems, tobacco, dental research, periodontal diseases, nicotine, oral health

## Abstract

Novel nicotine products, particularly electronic cigarettes (e-cigarettes), have become increasingly popular over the past decade. E-cigarettes are sometimes regarded as a less harmful alternative to tobacco smoking, and there is some evidence of their potential role as a smoking cessation aid. However, there are concerns about their health consequences, particularly in users who are not tobacco smokers, and also when used long term. Given the mode of delivery of these products, there is potential for oral health consequences. Over the past few years, there have been an increasing number of studies conducted to explore their oral health effects. In vitro studies have reported a range of cellular effects, but these are much less pronounced than those resulting from exposure to tobacco smoke. Microbiological studies have indicated that e-cigarette users have a distinct microbiome, and there is some indication this may be more pathogenic compared to nonusers. Evidence of oral health effects from clinical trials is still limited, and most studies to date have been small in scale and usually cross-sectional in design. Epidemiological studies highlight concerns over oral dryness, irritation, and gingival diseases. Interpreting data from e-cigarette studies is challenging, given the different populations that have been investigated and the continual emergence of new products. Overall, studies reveal potential oral health harms, underscoring the importance of efforts to reduce use in nonsmokers. However, in smokers who are using e-cigarettes as an aid to help them quit, the benefits of quitting tobacco smoking may outweigh any negative oral health impacts of e-cigarette use, particularly in the short term. Future research is needed to understand the clinical significance of some of the biological changes observed by following different cohorts of users longitudinally in carefully designed clinical studies and pragmatic trials supported by high-quality in vitro studies.

## Introduction

Tobacco smoking is a major risk factor for oral diseases such as oral cancer and periodontitis (US Surgeon General 2014). Dental professionals have an important role to play in providing smoking cessation advice and support to their patients who smoke and in considering this risk factor when planning and providing dental treatment. Nicotine is the main psychoactive, chemically addictive component in tobacco smoke. Yet, it is now widely accepted that nicotine is not responsible for the general health harms that result from smoking (National Institute for Health and Care Excellence 2013; Stratton et al. 2018). Nicotine has been used therapeutically as a smoking cessation aid for over 3 decades in the form of nicotine replacement therapies (NRTs), and high-quality evidence supports NRT effectiveness.

Between 2006 and 2009, a new category of nicotine products started to emerge, electronic cigarettes or e-cigarettes. There are now over 40 million users of e-cigarettes worldwide, and in 2019, the industry was estimated to be worth over US$19.3 billion per annum (British Broadcasting Corporation 2019). E-cigarettes generally contain 3 main categories of ingredients: a carrier solution (propylene glycol and/or vegetable glycerin), nicotine (although some e-cigarettes are nicotine free), and flavorings. E-cigarettes have proved to be controversial, and their potential risks and benefits have been extensively debated in many health and social care disciplines. From a smoking cessation perspective, there is clinical trial evidence that they are an effective tobacco cessation aid when used in a specialist environment (Liu et al. 2018; Hartmann-Boyce et al. 2020), but large population-based studies have presented conflicting conclusions (Beard et al. 2020; Pierce et al. 2020). To date, many of the clinical trials have been conducted on specific populations (e.g., dependent smokers, with high rates of social disadvantages, and motivated to quit) and in specialist environments with expert support. It is likely that the effectiveness of e-cigarettes demonstrated in these trials will not be replicated in “real-world” settings, and it is important that future trials evaluate this. The regulation of e-cigarettes varies around the world, but in the United States, e-cigarettes have yet to receive US Food and Drug Administration (FDA) approval as quit aids (Lavacchi et al. 2020). It is important for dental professionals to understand if e-cigarettes are an effective smoking cessation aid as this will have substantial impacts on oral health. However, it is also important to understand the standalone risks that e-cigarette use may present. The Table summarizes some of the potential merits and disadvantages of e-cigarettes.

**Table.** Summary of the Potential Merits and Disadvantages of E-Cigarettes.

Potential Merits	Potential Disadvantages
Potential effectiveness as a smoking cessation aid—clinical trial evidence reports that e-cigarettes are twice as effective as conventional nicotine replacement therapyPlausibly less health harms than conventional tobacco smoking	Effectiveness as cessation aid outside clinical settings (i.e., as consumer product) is unprovenAs a quit aid, concerns over long-term use (users appear to use for longer than other products such as nicotine replacement therapy)
Behavioral characteristics replicate habit—hand-to-mouth action, vapor production—for current smokersSeveral features valued by current tobacco smokers who would otherwise resist other cessation strategies: range of flavors and designs, nonmedicinal background and marketing, accessibility	Unknown long-term health impactsRegulatory approaches are varied around the world—rarely have e-cigarettes been regulated as strictly as medicinal productsMany features appeal to youth: range of flavors and designs, nonmedicinal background and marketing, accessibilityIn some regions, uptake by youth has been rapid and widespread; nicotine dependence common among youth users
	Concerns over health harms such as brain development in adolescence
	Some marketing and advertising have substantial youth appeal
	Dual use of e-cigarettes and conventional cigarettes together is common and may cause harms similar to conventional smoking alone

Tobacco smoking is responsible for considerable morbidity and mortality, with half of all smokers dying from a smoking-related disease such as cancer, respiratory disease, or vascular disease (Doll et al. 2004). The systemic health effects of e-cigarettes are still under investigation and the subject of some debate. It is widely accepted that e-cigarettes emit fewer toxicants than tobacco smoking (US Surgeon General 2014). A recent systematic review (Hartmann-Boyce et al. 2020), which included studies with a maximum 2-y follow-up, did not identify evidence of harmful effects caused by nicotine-containing e-cigarettes. Several other reviews have identified possible cardiovascular and respiratory harms (Gotts et al. 2019; Buchanan et al. 2020; Miyashita et al. 2020). Particular concerns have been raised about potential harms on the developing adolescent brain that require more investigation (US Surgeon General 2014).

The possible oral health effects of e-cigarettes have been vigorously debated in the dental literature over recent years. In this article, we review the evidence in 4 main areas: 1) basic science studies that evaluated cell lines and tissue cultures, 2) microbiological evidence from basic science and clinical research, 3) evidence from clinical studies evaluating oral health and smoking cessation (in dental settings), and 4) evidence from epidemiological studies. The topic of e-cigarettes can be emotive, which can create the risk of lack of equipoise in research studies. Likewise, the field has been subject to “hot stuff bias,” which refers to the concept that when a topic is new or fashionable, investigators may be less critical in their approach and journal reviewers and editors not as rigorous as they might otherwise be given the temptation to publish results (Catalogue of Bias Collaboration et al. 2017). In this review, we aspired to present a balanced and critical review of the current evidence on the oral health effects of e-cigarettes and make recommendations for future research.

## In Vitro Evidence

Toxicology studies have identified many components in e-cigarette aerosol (sometimes called vapor) that are hazardous to health, including nanoparticles, volatile organic compounds, carbonyls, heavy metals, and nicotine (Wang et al. 2019). Given that e-cigarette aerosol is inhaled, many studies have investigated effects on the respiratory system (Gotts et al. 2019) or the in vitro effects on oral cells and tissues (Yang et al. 2020). To date, at least 15 studies have investigated in vitro effects of e-cigarette exposure on 2-dimensional or 3-dimensional cell culture models involving normal, dysplastic, and cancerous oral keratinocytes, or gingival or periodontal ligament fibroblasts, including primary cells from human donors or established cell lines (reviewed by Yang et al. 2020).

Early studies tended to expose cells directly to e-liquids (Willershausen et al. 2014; Sancilio et al. 2016; Welz et al. 2016; Sancilio et al. 2017), giving an assessment of potential cellular effects. The studies reported cytotoxicity, which varied depending on the e-liquids used (Willershausen et al. 2014; Sancilio et al. 2016; Welz et al. 2016; Sancilio et al. 2017). Interestingly, none of these studies had a tobacco comparator. Unfortunately, this exposure method is not representative of the in vivo situation and often resulted in nonphysiological concentrations being studied. The use of cytotoxic levels of nicotine is an issue that has previously been identified with broader nicotine research (Holliday, Campbell, et al. 2019).

**Figure 1.**
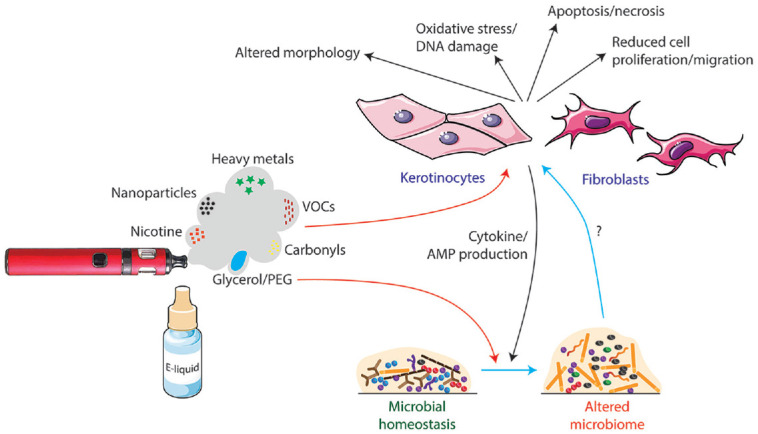
The potential mechanisms of the impact of e-cigarettes on oral health as presented by in vitro and microbiological studies to date. E-cigarettes contain a variety of bioactive agents that induce potentially damaging effects on cells such as human oral keratinocytes and periodontal fibroblasts in vitro. There is evidence that the glycerol or polyethylene glycol (PEG) carrier may be responsible for increased biofilm volume and alterations to the composition of the microbiome. It is not known how these changes affect host cells. Conversely, the induction of inflammation by e-cigarettes and release of cytokines and antimicrobial peptides (AMPs) into saliva may affect the oral microbiome. VOCs, volatile organic compounds. Parts of this image are adapted from Servier Medical Art (https://smart.servier.com).

Later work used e-cigarette aerosol extracts, prepared from custom-made extraction machines, added to the cell culture medium (Ji et al. 2016; Sundar et al. 2016; Yu et al. 2016; Ganapathy et al. 2017; Alanazi et al. 2018; Ji et al. 2019; Sun et al. 2019). This is closer to in vivo conditions, but it is hard to fully assess due to varied media composition resulting from different puffing protocols and extract dilutions. These studies reported a range of effects, including cytotoxicity, reduced cell proliferation and migration, increased apoptosis and inflammatory mediator production, and detection of oxidative damage such as protein carbonylation and DNA strand breaks (Fig. 1). Some of these studies used tobacco smoke extract as a comparator and consistently reported this to be more damaging (Yu et al. 2016; Ganapathy et al. 2017; Alanazi et al. 2018; Sun et al. 2019). Yu et al. (2016) found that tobacco smoke extract exposure was highly toxic, and they were only able to expose cells for 24 h without causing excessive cell death, whereas cells exposed to e-liquid extract survived for up to 8 wk, with new extract being added every 3 d.

More recent studies have exposed cells to e-cigarette aerosols directly, giving a more realistic exposure model. Rouabhia et al. (2017) reported altered cellular morphology, increased apoptosis/necrosis, and increased lactate dehydrogenase activity but did not include a tobacco comparator. Finally, Iskandar et al. (2019) employed a state-of-the art exposure system with organotypic tissue models and air-liquid interface system to compare e-cigarette vapor with air and tobacco smoke controls. The e-cigarette aerosols exposure induced cellular and molecular changes but at lower levels than exposure to diluted (3% to 24%) tobacco smoke. There were some useful insights into specific components of the e-liquid, with similar effects being seen between the carrier alone and when in combination with nicotine and flavorings.

It is important not to extrapolate directly from experimental results obtained in vitro to the clinical situation. For example, low-level changes in cell proliferation or apoptosis may not indicate an increased risk for oral cancer but could reflect the biological consequences of (naturally occurring) reestablishment of cellular homeostasis. Furthermore, reported statistically significant differences are not necessarily relevant in a biological or medical context. Comparative assessment between e-cigarettes and tobacco smoke, as well as between different e-cigarettes, such as different flavors, may be the most useful, complementing clinical studies that are unable to evaluate large numbers of parameters. Long-term exposure studies are lacking and are likely to be problematic due to the requirement for aseptic cell culture conditions.

In summary, although e-cigarette aerosol has been shown to cause abnormalities in oral cells in vitro, the significance of these biological effects in vivo is currently unclear. In the future, carefully designed and interpreted in vitro studies using standardized protocols that more closely mimic conditions during human use will be essential to improve understanding of the true effects of e-cigarettes on oral cell biology.

## Microbiological Evidence

Since most adult e-cigarette users are former tobacco smokers or dual users (those who continue to smoke), it is important to first understand the effects of smoking on the oral microbiome. Changes in the microbiome that are driven by smoking will potentially influence the baseline characteristics of e-cigarette users. However, the evidence regarding associations between tobacco smoking and the oral microbiome is evolving. For example, analysis of oral wash samples from 1,204 US adults identified depletion of Proteobacteria and enrichment for Firmicutes and Actinobacteria in smokers (Wu et al. 2016). By contrast, a smaller study that evaluated the microbiome at different sites in the mouth of 23 current smokers and 20 nonsmokers found little evidence for microbial shifts associated with smoking except at the buccal mucosa, where smokers had lower α-diversity (variation of microbes in a single sample) than nonsmokers (Yu et al. 2017). It is possible that changes are relatively subtle or highly variable and are not easily detected in small cohorts.

**Figure 2.**
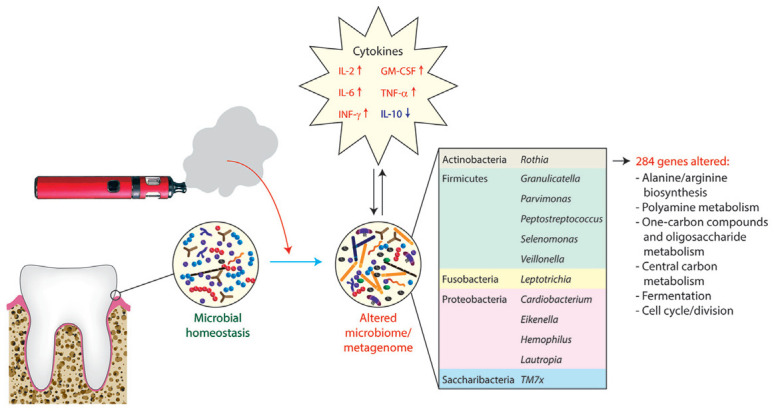
Impact of e-cigarette use on the oral microbiome and metagenome. Based on data from Ganesan et al. (2020), e-cigarette users (who self-reported not smoking tobacco) had increased α-diversity and enrichment of species within several genera of bacteria in subgingival dental plaque compared with nonsmokers who had never used e-cigarettes. The inset table shows genera containing species that were also elevated in saliva of e-cigarette users compared with either nonsmokers or cigarette smokers in a separate study (Pushalkar et al. 2020). The use of e-cigarettes was associated with changes in 284 genes, encoding a variety of metabolic pathways and other functions. Several proinflammatory cytokines were elevated in e-cigarette users including interleukin (IL)-2 and IL-6, granulocyte-macrophage colony-stimulating factor (GM-CSF), tumor necrosis factor α (TNF-α), and interferon-γ (IFN-γ), whereas the anti-inflammatory cytokine IL-10 was decreased.

There is recent evidence that the use of e-cigarettes may influence the profile of the oral microbiome toward a state that is distinct from that present in nonsmokers or tobacco smokers. For example, shotgun sequencing of pooled subgingival plaque samples of periodontally healthy e-cigarette users (without a history of smoking), smokers, and controls identified nearly 300 genes that were enriched in e-cigarette users, encoding components of pathways such as arginine and alanine biosynthesis, metabolism of polyamines, 1-carbon compounds and (oligo)saccharides, central carbon metabolism, fermentation, and cell cycle/cell division (Ganesan et al. 2020). Taxonomically, the microbiome in e-cigarette users had greater α-diversity and higher levels of several phyla and genera, including Actinobacteria, certain Firmicutes (including *Selenomonas* and *Veillonella*), Fusobacteria, Proteobacteria, and Saccharibacteria (Fig. 2). By contrast, Gram-negative obligate anaerobes were more strongly enriched in the subgingival biofilm of smokers. Similarly, the salivary microbiome appears to be distinct in e-cigarette users with no history of smoking compared with smokers or never-smokers. Increased β-diversity (variation of microbial communities between samples) and higher levels of *Actinomyces*, *Porphyromonas*, and *Veillonella* were observed in e-cigarette users (Pushalkar et al. 2020). Conflictingly, an early pilot oral microbiome study of 10 e-cigarette users found no statistically significant differences from 10 nonsmokers (Stewart et al. 2018). In 2 of the above studies, exhaled carbon monoxide levels were slightly higher in e-cigarette users than in the nonsmoker/never-user control group (Stewart et al. 2018; Pushalkar et al. 2020). Although differences were not statistically significant, it is possible that some of the e-cigarette cohort may also have smoked tobacco at some point prior to sampling. No data on carbon monoxide or salivary analyses were given by Ganesan et al. (2020). Overall, current evidence suggests that e-cigarette usage is associated with changes in the microbiome that are distinct from smoking. However, larger-scale studies in different populations around the world with careful monitoring of smoking status are needed to clarify the associations between the oral microbiome and usage of e-cigarettes.

In vitro studies have shown that e-cigarette exposure has a relatively modest effect on the growth and survival of oral streptococci compared with cigarette smoke (Cuadra et al. 2019). However, exposure to e-cigarette aerosol resulted in transcriptional changes in the related species *Streptococcus pneumoniae* without affecting virulence properties (Bagale et al. 2020). Similarly, transcriptional profiles of defined mixed-species biofilms shifted in response to e-cigarette exposure (Ganesan et al. 2020). For example, pathways related to sialic acid metabolism were modulated in a variety of oral streptococci as well as *Veillonella parvula*, *Actinomyces naeslundii*, and *Neisseria mucosa*. Genes encoding functions related to biofilm matrix production and cell wall biosynthesis were also upregulated in biofilms. Biofilms exposed to e-cigarette aerosol were rich in matrix material and had greater surface area and volume than control biofilms. It is noteworthy that these effects appeared to be due to components of the e-cigarettes other than nicotine, most likely sugar alcohols such as glycerol or polyethylene glycol that are used as vehicles in e-cigarettes and can be a source of nutrients for microorganisms (Ganesan et al. 2020). It is also possible that e-cigarettes modulate the oral microbiome indirectly. Indeed, they have been shown to affect levels of antimicrobial proteins and cytokines in saliva (Cichońska et al. 2019; Ganesan et al. 2020). In vitro, e-cigarette exposure increased proinflammatory cytokine production by premalignant and malignant cell lines, as well as accelerated infection by oral bacteria (Pushalkar et al. 2020). The evidence to date, therefore, suggests that e-cigarette use has quantifiable effects on the oral microbiome. However, the clinical implications of these effects and risk for oral disease have yet to be determined, and a significant limitation in this area is the lack of longitudinal studies. Furthermore, particularly within the context of periodontal diseases, which are characterized by significant dysbiosis of the subgingival microbiome, it may be challenging to reproducibly identify impacts of e-cigarette use.

## Evidence from Clinical Studies

There are many challenges in conducting clinical research using e-cigarettes. Hence there are few studies to date, and most of these have significant limitations. Major barriers for clinical studies have included the regulatory environment and difficulties securing funding. For example, some research funders have not allowed their funds to cover smoking cessation studies using e-cigarettes or, if they allow them, they may not fund the provision of e-cigarette products and supplies to participants, meaning that researchers have to obtain additional funding to cover these costs.

A recent systematic review (Yang et al. 2020) identified 65 clinical studies of the oral health impacts of e-cigarettes; most of these were observational and cross-sectional in design. An example of one of the earlier studies was a cross-sectional investigation of smokers, e-cigarette users, and nonusers (*n* = 65) in which cytologic examination of oral mucosa scrapings was performed (Franco et al. 2016). These authors found that cell damage was significantly decreased in the e-cigarette group (as compared to current smokers), being similar to that seen in healthy controls (never smokers) and concluded that e-cigarettes were safe for oral cells.

Given the extensive evidence linking tobacco smoke with increased risk for periodontitis and peri-implant disease, it is natural that the impacts of e-cigarettes on these conditions have been among the most studied areas. Overall, evidence suggests that the risk of periodontal disease associated with e-cigarette use is less than that associated with tobacco smoking but more than that seen in nonsmokers. Whereas a spectrum of risk for periodontitis and peri-implantitis exists from nonsmokers to smokers, it is not possible from the current evidence base to confidently locate e-cigarette users within this. However, a number of studies have reported clinical improvements in those with preexisting periodontal disease when participants quit smoking and switched to using e-cigarettes, with and without periodontal therapies being provided (Javed et al. 2017; ALHarthi et al. 2019; BinShabaib et al. 2019). There are less data available for peri-implantitis, although cross-sectional studies have suggested effects are less harmful than those seen in smokers but more than those in nonsmokers (ArRejaie et al. 2019).

A pilot study (Wadia et al. 2016) that evaluated gingival bleeding in e-cigarette users is often cited in reviews (ArRejaie et al. 2019; Atuegwu et al. 2019) or public health documents (Stratton et al. 2018) as evidence of increased bleeding with e-cigarette use. The study, which followed 18 smokers who were asked to switch to an e-cigarette for 2 wk, observed an increase in bleeding on probing during the time of e-cigarette use. However, whether this was a direct result of switching to an e-cigarette or was simply the commonly observed clinical finding of a transient increase in gingival bleeding when a smoker quits (Nair et al. 2003) remains unknown.

Of the 8 randomized controlled trials (RCTs) identified by Yang et al (2020), 7 took place in nondental settings (collecting oral/throat health parameters as ancillary findings, usually self-reported). A range of oral health outcomes were reported, with some studies finding more frequent complaints in e-cigarette users and others reporting fewer. One RCT was conducted in the dental setting and reported more detailed oral health measures (Holliday, Preshaw, et al. 2019). Although this RCT was pilot in design and hence not powered to provide definitive findings for smoking cessation or oral health outcomes, the authors reported descriptively that those in the e-cigarette group had a higher quit rate than those in the control group, and there were similar changes in oral health–related outcomes, including periodontal health parameters, oral dryness, and oral health quality of life between the 2 groups. Interestingly, there were 5 episodes of ulceration or soreness reported during the 6-mo follow-up period, all occurring in the e-cigarette group. The pilot data from this study have informed the design of a definitive multicenter RCT, recently funded by the UK National Institute for Health Research, which plans to recruit 1,460 smokers across 56 dental practices (HTA—NIHR129780 2020).

In summary, the clinical evidence is limited and challenging to interpret but suggests that e-cigarettes are less harmful to oral health than tobacco cigarettes and might be an effective cessation aid in dental settings.

## Epidemiological Evidence

Among prominent challenges in epidemiologically studying the oral health effects of e-cigarettes is that the most prevalent oral diseases are chronic conditions that develop over the life course, while e-cigarette use gained substantial popularity only within the past 5 to 10 y. In addition, most adult e-cigarette users are current or former cigarette smokers, making it difficult to separate any e-cigarette effects from oral health problems caused by prior or current conventional smoking. Furthermore, given that periodontal disease risk increases with age, the much higher e-cigarette use prevalence among younger individuals not only confounds associations but also might obscure health effects not manifest until later life. Currently, most epidemiologic studies of e-cigarettes and oral health rely on cross-sectional designs and self-reported health measures, which limits evidence quality. It may require decades to estimate precisely the population-level impact of e-cigarettes on oral health, but emerging associations suggest potential for harm.

Numerous case reports demonstrate the presence of oral mucosal, tongue, or ulcerative lesions in patients who use e-cigarettes, as recently reviewed (Yang et al. 2020). The population prevalence of such lesions and whether directly caused by e-cigarette use cannot be inferred from case reports. However, a number of cases of maxillofacial trauma are readily attributable to explosive e-cigarette device failures, including intraoral burns and alveolar fractures. While these traumatic injuries are notable for dental practice, such explosions are presumably avoidable with quality manufacturing standards.

A number of studies not explicitly designed to assess oral health have identified dry mouth and mouth or throat irritation as common symptoms among e-cigarette users. In a systematic review of 11 studies that assessed e-cigarettes as potential smoking cessation aids, cough or mouth/throat irritation was the most commonly reported group of adverse events, occurring in up to 39% of participants (Liu et al. 2018). This corroborates a US national telephone survey in which cough (40%) and dry or irritated mouth or throat (31%) were the most common of 6 recorded symptoms among adult e-cigarette ever-users (King et al. 2019).

In nationally representative cross-sectional studies, associations between e-cigarette use and worse oral health have been reported, including after confounding adjustment. Among children in South Korea, mouth pain and broken or cracked teeth were associated with e-cigarette use (Cho 2017). In the United States, parent report of “dental problems” was more common among children who used both e-cigarettes and conventional cigarettes versus tobacco nonusers (Akinkugbe 2019). Among US adults, daily e-cigarette users were slightly more likely to report ever having a tooth removed for gum disease or decay than were never-users (56% vs. 51%) (Huilgol et al. 2019). Similarly, ever being diagnosed or treated for gingival disease was higher among e-cigarette users than tobacco never-users (Vora and Chaffee 2019). In South Korea, the odds of examiner-measured periodontal disease were approximately double for both e-cigarette users and conventional cigarette smokers versus nonusers (Jeong et al. 2020).

There are few sources of longitudinal evidence, and epidemiologic data from outside the United States and South Korea are currently lacking. In 1 US study, adults who used e-cigarettes in 3 consecutive annual survey waves had higher odds at the third wave of self-reported gingival disease and bone loss around teeth (Atuegwu et al. 2019).

## Synthesis of the Evidence

Throughout the 4 sections of this review, we have explored the many challenges of studying the oral health consequences of e-cigarette use and limitations of the current evidence base. While a number of studies suggest that oral health harms are plausible, it remains difficult to quantify the potential risks to patients.

A common challenge is the heterogeneity of the product category, given that there are hundreds of different e-cigarette devices, thousands of e-liquid and flavor formulations, and a constantly changing market. Hence, generalizing the findings of a study on a single product to the whole e-cigarette universe can be challenging. User choice is also another particularly unique aspect of e-cigarette interventions and does not easily fit within the tightly controlled designs of clinical trials. Accordingly, we consider that clinical trials (including RCTs) are best designed to be at the pragmatic end of the explanatory-pragmatic spectrum (Loudon et al. 2015). For example, this might involve providing an e-cigarette starter kit and advice, then allowing participants to source their own supplies thereafter (and accepting that they may switch flavors or nicotine concentrations over time). Findings from clinical trials are best presented and interpreted in this broad sector-wide context. In vitro studies will also play a useful role in assessing the relative effects between the different parameters (nicotine concentrations, flavors, carrier solutions, device settings, and puffing patterns), which would be impractical to evaluate in clinical studies.

An important consideration when appraising studies in this field relates to perspective (influenced by participant groups) and the comparisons that are made. There are 2 very different populations that are typically studied: 1) never-smoker e-cigarette users with no preexisting oral health conditions, perhaps representing a young person who takes up vaping, having never smoked tobacco previously; or 2) tobacco smokers who are using an e-cigarette to quit or for other reasons and who will often have preexisting oral disease such as periodontitis. This latter group may also be “dual users” in that they may be using both tobacco cigarettes and an e-cigarette at the same time. Most of the evidence suggesting harm from e-cigarettes comes from studies on the first group (healthy individuals), highlighting the importance of public health efforts to reduce use in nonsmokers. For the latter group (smokers with existing oral diseases), observing and measuring the oral health impacts of e-cigarettes is complex; any minor changes can be masked by the preexisting disease status and concurrent changes in tobacco use (e.g., increased e-cigarette use and decreased tobacco use). Hence, studies should be interpreted cautiously, and it is important to not overgeneralize findings from specific populations.

It is clear from the existing evidence that tobacco smoking has major negative impacts on oral health (US Surgeon General 2014). Dental professionals are potentially in a powerful position to provide smoking cessation advice and support, and this is known to be well received by patients (Holliday et al. 2020). There are a range of proven behavioral and pharmacological interventions available. E-cigarettes may become a useful addition to this armamentarium, pending further study. The evidence base suggests that e-cigarette use has the potential to cause oral health consequences, particularly in those with no existing oral disease, and efforts in preventing use in nonsmokers are therefore critical. However, for smokers with existing oral diseases, the major benefits of quitting tobacco smoking are likely to outweigh any negative impacts from e-cigarette use, particularly over the short term. Hence, if a smoker is keen to use an e-cigarette as part of a quit attempt, we consider (according to currently available evidence) that they should not be discouraged from doing so based on oral health concerns. However, we further consider that such use of e-cigarettes should be as part of a structured quit attempt and participating individuals offered additional cessation support, such as professional counseling and pharmacotherapy as appropriate to the situation.

Along with the existing challenges we have discussed, there are a range of future hurdles for researchers in this field, not least being the fact that new products are continually emerging or gaining popularity, such as heated tobacco products and dissolvable tobacco and nicotine pouches. Future research should focus on these products as they emerge.

## Conclusions

Studying the oral health consequences of e-cigarette use is challenging given the changing nature of the products and difficulties of identifying potential e-cigarette effects in patients with a past or current history of combustible tobacco use. While the evidence base is limited, it does suggest that there are potential oral health harms associated with e-cigarette use. For those using e-cigarettes as a tobacco quit aid, the evidence of oral health impacts is uncertain and complicated by the substantial oral health changes that occur when users quit tobacco smoking. There is a clear need for further well-conducted studies in this field. Those areas that have the strongest potential to benefit patients are understanding the oral health consequences in nonsmokers who initiate e-cigarette use, establishing the effectiveness of e-cigarettes as a tobacco quit aid (especially within the dental setting), and understanding any impacts on periodontal health in smokers who switch to e-cigarettes.

## Author Contributions

R. Holliday, P.M. Preshaw, contributed to conception and design, drafted and critically revised the manuscript; B.W. Chaffee, N.S. Jakubovics, R. Kist, contributed to design, drafted and critically revised the manuscript. All authors gave final approval and agree to be accountable for all aspects of the work.
